# Prevalence and clinical manifestations of gastro-oesophageal reflux-associated chronic cough in the Japanese population

**DOI:** 10.1186/1745-9974-3-1

**Published:** 2007-01-08

**Authors:** Hisako Matsumoto, Akio Niimi, Masaya Takemura, Tetsuya Ueda, Masafumi Yamaguchi, Hirofumi Matsuoka, Makiko Jinnai, Kazuo Chin, Michiaki Mishima

**Affiliations:** 1 Department of Respiratory Medicine, Kyoto University, Kyoto, Japan

## Abstract

Gastro-oesophageal reflux (GOR) is one of the most common causes of chronic cough in Western countries, responsible for 10 to 40% of cases. In Japan, however, GOR-associated chronic cough (GOR-CC) has been rarely reported and its clinical manifestation including frequency of concomitant reflux laryngitis is poorly known.

We have analyzed prevalence and clinical characteristics of patients who were diagnosed as having GOR-CC among adult patients with chronic cough (≥ 8 weeks) who visited our asthma and cough clinic over a period of 19 months. Diagnosis of GOR-CC was based on the response of coughing to a proton-pump inhibitor (lansoprazole™) and/or positive results of 24 h ambulatory esophageal pH monitoring. Laryngeal involvement was based on symptoms or objective diagnosis by specialists.

GOR-associated chronic cough was diagnosed in 7.1% (8 of 112) of chronic cough patients. In addition to the demographic data which were consistent with the characteristics of patients with GOR-CC in the Western populations, including gender (6 females), age (mean ± SE, 56.9 ± 5.8 years), duration of cough (9.9 ± 3.3 months), lack of gastrointestinal symptoms (3 of 8) and complication with other causes of cough (5 of 8), we found the standard range of body mass index (23.9 ± 1.5 kg/m^2^) and high incidence of concomitant reflux laryngitis (5 of 8) in the present 8 patients. Among 4 patients who could stop treatment with temporal resolution of cough, cough recurred in 3 patients, 1 week to 8 months after the discontinuation.

In conclusion, GOR-CC is a less frequent cause of chronic cough in Japan than in Western countries. Signs or symptoms of laryngitis may be important as clues to suspicion of GOR-CC.

## Findings

Despite the established evidence that gastro-oesophageal reflux (GOR) causes 10 to 40% of chronic cough [1], and the prevalence of GOR-associated chronic cough (GOR-CC) is increasing in the Western populations [2], this condition has been rarely reported in Japan and its clinical manifestation is not well characterized. Only one case among 37 patients with chronic dry cough was diagnosed as having GOR-CC in our previous study carried out in the mid '90s [3]. Our present study is concerned with the rising number of cases of GOR-CC in Japan and of concomitant reflux laryngitis which is another major extra-oesophageal manifestation of GOR.

We studied 112 consecutive adult patients with chronic cough (≥ 8 weeks) who newly visited the asthma and cough clinic of Kyoto University Hospital from June 2002 to December 2003. Diagnostic investigations included questionnaire, physical examination, blood tests, chest and sinus radiographs, pulmonary function, airway responsiveness and cough sensitivity tests, and sputum induction. Diagnosis of GOR-CC was on the basis of response to 8 week-course of a proton-pump inhibitor (PPI, lansoprazole™) and/or positive results of 24 h ambulatory esophageal pH monitoring (pH Digitrapper MarkII Gold 6,200, Synetics Medical Comp., Sweden) [4]. Laryngeal involvement was based on symptoms or objective diagnosis by specialists; laryngeal irritation, globus sensation, or signs of laryngeal inflammation. Diagnosis of cough variant asthma (CVA) was based on the following criteria; an isolated chronic cough without dyspnea or wheezing not audible on auscultation, airway hyperresponsive to methacholine and symptomatic improvement of coughing with the use of inhaled beta-_2 _agonists, sustained release theophylline or both, no past history of asthma, or upper respiratory tract infection within the past 8 weeks [5]. When patients did not undergo methacholine test due to failure of informed consent or presented normo-responsive result but responded to bronchodilator therapy, they were diagnosed as having probable CVA[6]. Diagnosis of sinobronchial syndrome was made on a positive result of sinus images and improvement of cough as well as the symptom related to chronic sinusitis with antibiotics [7,8]. Diagnosis of atopic cough was made according to the criteria proposed by Japanese Cough Research Society [6,9]. If examinations and intensive therapeutic trials for GOR-CC, CVA, sinobronchial syndrome, and atopic cough including inhaled corticosteroids and anti-reflux treatment were failed, the chronic cough was considered unexplained (idiopathic). All the chronic cough patients showed normal chest radiographs.

Causes of chronic cough of the 112 patients were as follows; 38 CVA, 24 probable CVA (12 patients did not undergo airway responsiveness test and 12 presented normo-responsive results but responded to bronchodilator therapy), 17 atopic cough, 9 sinobronchial syndrome, 8 GOR-CC, 7 postinfectious chronic cough, 2 other miscellaneous conditions, 4 unexplained cough. Twelve patients were lost before diagnosis was made. Nine patients had multiple conditions. There were 15 ex-smokers, and 2 current smokers. No patients had ACE inhibitor associated chronic cough. The 8 patients (7.1%) with GOR-CC were diagnosed on the basis of response to the PPI (n = 7) and/or positive results of 24 h ambulatory esophageal pH monitoring (n = 4). One patient who complained of chronic cough and heartburn but did not respond to the PPI showed esophageal pH more than 7 in 66.9% of the 24 h monitoring period, and was diagnosed as having GOR-CC due to alkaline regurgitation.

Demographics of the 8 patients were presented in Table 1. Mean body mass index (BMI) was classified as normal at 23.9 (range, 19.4–28.0) kg/m^2^, which was not significantly different from that of the general population in the present study (23.3 kg/m^2^, 16.5–36.3 kg/m^2^). Frequent association of reflux laryngitis (5 of 8) was observed. Two patients complained of temporal association of coughing and heartburn. Five patients were complicated with other causes of chronic cough; 3 with CVA/probable CVA on inhaled corticosteroids or an anti-leukotriene receptor antagonist, 1 with sinobronchial syndrome on low dose of macrolide, 1 with atopic cough on an anti-histamine receptor antagonist. Airway responsiveness was tested in 4 patients among whom 2 with complication of CVA showed hyperresponsiveness. Cough sensitivity was examined in 2 patients; one complicated with atopic cough had hypersensitivity, another with CVA did not. All were non-smokers and produced minimal amount of sputum or none.

**Table 1:** Patients' characteristics

Age (years)	56.9 ± 5.8
Gender (male/female)	2/6
Cough duration (months)	9.9 ± 3.3
Body mass index (kg/m^2^)	23.9 ± 1.5
FEV_1 _(%predicted)	98.5 ± 5.3
Intraesophageal symptoms* (yes/no)	5/3
Reflux laryngitis (yes/no)	5/3

Values are expressed as mean ± SE. * heart burn and acid reflux.

In 7 patients, their coughing with or without laryngeal symptoms was alleviated and ceased within a few days after initiation of PPI. However, in 3 of 4 patients who had stopped treatment with resolution of cough, coughing recurred 1 week to 8 months later. Coughing, laryngitis and heart burn observed in one patient with alkaline regurgitation subsided without any treatment.

In the present study, GOR-CC was a less frequent cause of chronic cough than in the studies of the Western populations [1,2], although there was a small increase when comparing with our previous study [3]. The low prevalence of GOR-CC in the present study might be biased by the studied population in a university hospital, but may reflect low prevalence of GOR in the general population in Japan where 21 to 27% of the general population are overweight (BMI ≧ 25 kg/m^2^) [10] and endoscopically diagnosed and/or symptomatic GOR is reported to be 16% and 18% in population based studies [11,12]. This is in contrast with the finding in Western countries where 34 to 78% of the general population are overweight [10] and 21 to 59% of the general population have symptomatic GOR [13].

In one patient who complained of cough and heartburn, and presented laryngitis and frequent alkaline regurgitation, the cough had ceased spontaneously as well as heartburn and laryngitis. Although there are no diagnostic criteria for alkaline regurgitation, the patient's cough was clinically considered caused by GOR-CC due to alkaline regurgitation.

The present study has shown frequent association of reflux laryngitis and GOR-CC. To date, frequency of this association has not been clarified [2]. In a study of extra-oesophageal manifestations in GOR, laryngeal manifestations are observed in 10.4% of patients with GOR and are significantly related to higher age, longer GOR duration and obesity [14], although these features were not the cases for the 5 patients with average age of 47.4 (28–59) years and BMI of 22.4 (19.2–28) kg/m^2 ^in the present study. Except the frequent incidence of concomitant reflux laryngitis and standard range of BMI, characteristics of the present patients including complication with other causes of chronic cough were consistent with those in the previous studies of GOR-CC in the Western populations [1,4,15,16]. Since chronic cough *per se *can be a trigger of GOR possibly through increased transdiaphragmatic pressure and transient lower oesophageal sphincter relaxation [17], GOR-CC should be considered when cough remains despite the institution of specific treatment to other causes of cough.

We conclude that GOR-CC is a less frequent cause of chronic cough in Japan than in Western countries. The presence of reflux laryngitis may be an important clue to suspicion of GOR-CC.

## Abbreviations

BMI = body mass index

CVA = cough variant asthma

GOR = gastro-oesophageal reflux

GOR-CC = GOR-associated chronic cough

## Footnote

The ethics committee of our institution approved the study protocol and a written informed consent was obtained from each participant.

## Financial support

This study was supported by Takeda Pharmaceutical Company ltd.

## Competing interests

The author(s) declare that they have no competing interests.

## Authors' contributions

H Matsumoto conceived of the study, participated in its design, acquisition, and interpretation of data, and drafted the manuscript.

AN conceived of the study, participated in its design, contributed to data interpretation.

MT participated in acquisition of data

TU participated in acquisition of data

MY participated in acquisition of data

HM participated in acquisition of data

MJ participated in acquisition of data

KC contributed to data interpretation

MM contributed to data interpretation

## References

[B1] Fontana GA, Pistolesi M (2003). Cough. 3: chronic cough and gastro-oesophageal reflux. Thorax.

[B2] Irwin RS (2006). Chronic cough due to gastroesophageal reflux disease: ACCP evidence-based clinical practice guidelines. Chest.

[B3] Matsumoto H, Niimi A, Satou S, Kishi K (2000). [Chronic cough caused by gastroesophageal reflux]. Nihon Kokyuki Gakkai Zasshi.

[B4] McGarvey LP, Heaney LG, Lawson JT, Johnston BT, Scally CM, Ennis M, Shepherd DR, MacMahon J (1998). Evaluation and outcome of patients with chronic non-productive cough using a comprehensive diagnostic protocol. Thorax.

[B5] Niimi A, Amitani R, Suzuki K, Tanaka E, Murayama T, Kuze F (1998). Eosinophilic inflammation in cough variant asthma. Eur Respir J.

[B6] The committee for The Japanese Respiratory Society guidelines for management of cough (2006). Prolonged and chronic cough. Respirology.

[B7] Niimi A Geography and cough aetiology. Pulmonary Pharmacology & Therapeutics.

[B8] Fujimura M, Mizuguchi M, Nakatsumi Y, Mizuhashi K, Sasaki S, Yasui M (2002). Addition of a 2-month low-dose course of levofloxacin to long-term erythromycin therapy in sinobronchial syndrome. Respirology.

[B9] Fujimura M, Ogawa H, Nishizawa Y, Nishi K (2003). Comparison of atopic cough with cough variant asthma: is atopic cough a precursor of asthma?. Thorax.

[B10] WHO::Global Database on Body Mass Index [www.who.int/bmi/index.jsp].

[B11] Furukawa N, Iwakiri R, Koyama T, Okamoto K, Yoshida T, Kashiwagi Y, Ohyama T, Noda T, Sakata H, Fujimoto K (1999). Proportion of reflux esophagitis in 6010 Japanese adults: prospective evaluation by endoscopy. J Gastroenterol.

[B12] Mishima I, Adachi K, Arima N, Amano K, Takashima T, Moritani M, Furuta K, Kinoshita Y (2005). Prevalence of endoscopically negative and positive gastroesophageal reflux disease in the Japanese. Scand J Gastroenterol.

[B13] Heading RC (1999). Prevalence of upper gastrointestinal symptoms in the general population: a systematic review. Scand J Gastroenterol Suppl.

[B14] Jaspersen D, Kulig M, Labenz J, Leodolter A, Lind T, Meyer-Sabellek W, Vieth M, Willich SN, Lindner D, Stolte M, Malfertheiner P (2003). Prevalence of extra-oesophageal manifestations in gastro-oesophageal reflux disease: an analysis based on the ProGERD Study. Aliment Pharmacol Ther.

[B15] Irwin RS, Zawacki JK, Curley FJ, French CL, Hoffman PJ (1989). Chronic cough as the sole presenting manifestation of gastroesophageal reflux. Am Rev Respir Dis.

[B16] Kiljander TO, Salomaa ER, Hietanen EK, Terho EO (2000). Chronic cough and gastro-oesophageal reflux: a double-blind placebo-controlled study with omeprazole. Eur Respir J.

[B17] Ing AJ, Breslin AB (1997). The patient with chronic cough. Med J Aust.

